# Neurodevelopmental disorders and cancer networks share pathways, but differ in mechanisms, signaling strength, and outcome

**DOI:** 10.1038/s41525-023-00377-6

**Published:** 2023-11-04

**Authors:** Bengi Ruken Yavuz, M. Kaan Arici, Habibe Cansu Demirel, Chung-Jung Tsai, Hyunbum Jang, Ruth Nussinov, Nurcan Tuncbag

**Affiliations:** 1 Graduate School of Informatics, Middle East Technical University, Ankara, 06800 Turkey; 2 Cancer Innovation Laboratory, National Cancer Institute, Frederick, MD 21702 USA; 3 Graduate School of Sciences and Engineering, Koc University, Istanbul, 34450 Turkey; 4 Computational Structural Biology Section, Frederick National Laboratory for Cancer Research in the Cancer Innovation Laboratory, National Cancer Institute, Frederick, MD 21702 USA; 5 Department of Human Molecular Genetics and Biochemistry, Sackler School of Medicine, Tel Aviv University, Tel Aviv, 69978 Israel; 6 Chemical and Biological Engineering, College of Engineering, Koc University, Istanbul, Turkey; 7 School of Medicine, Koc University, Istanbul, 34450 Turkey; 8 Koc University Research Center for Translational Medicine (KUTTAM), Istanbul, Turkey

**Keywords:** Computational biology and bioinformatics, Systems biology

## Abstract

Epidemiological studies suggest that individuals with neurodevelopmental disorders (NDDs) are more prone to develop certain types of cancer. Notably, however, the case statistics can be impacted by late discovery of cancer in individuals afflicted with NDDs, such as intellectual disorders, autism, and schizophrenia, which may bias the numbers. As to NDD-associated mutations, in most cases, they are germline while cancer mutations are sporadic, emerging during life. However, somatic mosaicism can spur NDDs, and cancer-related mutations can be germline. NDDs and cancer share proteins, pathways, and mutations. Here we ask (i) exactly which features they share, and (ii) how, despite their commonalities, they differ in clinical outcomes. To tackle these questions, we employed a statistical framework followed by network analysis. Our thorough exploration of the mutations, reconstructed disease-specific networks, pathways, and transcriptome levels and profiles of autism spectrum disorder (ASD) and cancers, point to signaling strength as the key factor: strong signaling promotes cell proliferation in cancer, and weaker (moderate) signaling impacts differentiation in ASD. Thus, we suggest that signaling strength, not activating mutations, can decide clinical outcome.

## Introduction

Neurodevelopmental disorders (NDDs) encompass a broad spectrum of abnormalities in brain development that can affect cognition, communication, behavior, and motor functions^1^. Genetics and risk factors can play a role during different stages of brain development^2^. NDDs may originate from dysregulation of neuron differentiation during synapse formation and maturation, including formation of specific synaptic contacts, or during other complex processes, such as emergence from progenitor cells, neuron phenotypic specification and migration. Disruption of convergent pathways, including mitochondrial/metabolic processes, PI3K/mTOR, MAPK, and Wnt signaling was also suggested to explain the etiology of NDDs^3^. NDDs and cancer are highly complex diseases caused by impairments in cellular processes such as cell growth, proliferation, and differentiation. This challenging complexity has led to investigations into how their genetics, cellular environment, and signaling pathways are converging to express their distinct phenotypic outcomes^4–14^. Cancer results from gene alterations that provide cells growth advantage. Numerous studies focused on the connection between the mutations—germline, de novo, or somatic—and cancer^15–20^. The number of studies related to NDDs increased, though still lagging behind those of cancer, far from reaching the same level. Qi et al. observed that cancer driver genes are more significantly enriched in germline damaging de novo variants among patients with NDDs as compared to non-drivers^7^. Additionally, a comprehensive analysis on 219 cancer-related genes and de novo mutations from 16,498 patients with NDDs (including ASD, congenital heart disease, and intellectual disability) revealed that de novo mutations are located significantly more in cancer-related genes compared to control samples^21^. In another study focusing on ASD, an evolutionary action method identified missense de novo variants that most likely contribute to the etiology of the disorder^22^.

Despite seemingly differing from processes associated with the emergence of cancer, data indicate that NDDs and cancer are related. One recent hypothesis is that immunity can be a common factor connecting these two phenotypes since the immune and nervous systems coevolve as the embryo develops. The outcomes, cancer or NDDs, reflect the different cell consequences, primarily proliferation in cancer and differentiation in NDDs. Cell proliferation requires a stronger signal than differentiation does. This further suggests that in addition to proteins in the major signaling pathways, transcription factors (TFs), and chromatin remodelers, which govern chromatin organization, are key agents in NDDs. Gene accessibility influences the lineage of specific brain cell types at specific embryonic development stages^23^.

Recent epidemiological studies on large cohorts of NDD patients demonstrated an increased risk for cancer compared to the general population. In one study, a standardized incidence ratio model was applied to a cohort of 8438 patients with autism retrieved from the Taiwan National Health Insurance database during 1997-2011. The researchers reported an increased cancer risk for males and young adults. The occurrence of the genitourinary system and ovary cancers was observed to be higher than expected^24^. A population-based study among 2.3 million individuals from Nordic countries during 1987–2013 revealed that ASD patients with co-morbid conditions, such as intellectual disability and birth defects, had a higher risk than the general population, while ASD alone was not significantly linked to a higher risk of cancer^25^. A correlation between autism and cancer rates with shared risk factors was also pointed out^26^. Another cohort study proposed that patients with bipolar disorder and their unaffected siblings have a higher risk of developing breast cancer compared to normal control groups^27^. The association between brain, hepatocellular, and lung cancers among people with epilepsy was manifested by animal experiments, genotoxicity studies, and epidemiological observations. Possible underlying mechanisms have also been suggested^28,29^. Risk of testicular cancer was increased among patients with NDDs or other psychiatric disorders which is observed in a case-control study^30^.

NDD data has expanded recently, particularly de novo mutation data obtained by trio-sequencing and publicly available databases. However, it is still not as prevalent as the whole exome/genome sequencing data for cancer^31,32^. 32,991 de novo variants obtained from 23,098 trios are deposited in denovo-db^31^. According to the database definition, de novo mutations are germline de novo variants present in children but not in their parents. The Deciphering Developmental Disorders (DDD) Study provides detailed genotype-phenotype information for 14,000 children with developmental disorders, and their parents from the UK and Ireland. Additionally, there are some knowledge databases with curated sets of genes and variants associated with one/multiple neurodevelopmental disorders or cancer^33,34^. At the same time, despite epidemiology suggesting a positive correlation between NDDs and either overall or site-specific cancer risk^27,35–37^, not all epidemiological findings agree^38,39^. Some studies even reported a lower cancer incidence rate for NDD patients^40–42^. These discrepancies may result from factors such as genetic backgrounds, environmental effects, as well as diagnosis at an already advanced cancer stage especially in NDDs such as intellectual delay, autism, and schizophrenia.

With NDDs and cancer sharing multiple features on different biological levels^6,7,43,44^, here we aim to shed light on their possible connection. We expect that these will help us understand the challenging question of how expression levels and mutations in the same pathways, and even the same proteins, including TFs and chromatin remodelers, can lead to NDDs versus cancer, with vastly different phenotypic presentations. Especially, we aim to discover the features deciding the major outcome. We address these daunting goals by comprehensively leveraging mutations, transcriptomic data, and protein-protein interaction (PPI) networks. We compare the effects of mutations on the pathogenicity of commonly mutated genes in NDDs and cancer. To evaluate the pathway-level properties of NDDs and cancer, we reconstruct the disease-specific networks of autism spectrum disorder (ASD) and breast cancer and identify common TFs.

Here, we use de novo mutations in ~8000 samples with NDDs from denovo-db and somatic mutations of ~10,000 tumor samples from The Cancer Genome Atlas (TCGA). Our large-scale analysis led us to conclude that networks of NDDs and cancer can have shared proteins and pathways that differ in signaling strength, mechanisms, and outcomes. This conclusion is in line with our premise that cell-type-specific protein expression levels of the mutant protein, and other proteins in the respective pathway and their regulators, the timing of the mutations (embryonic or sporadic during life), and the absolute number of molecules that the mutations activate can determine the pathological phenotypes, cancer and (or) NDDs. Our thesis is that these define the signaling strengths. In cancer, the major impact is on cell proliferation, while in NDDs it is on differentiation.

## Results

### NDD versus cancer mutations and networks data

**Fig. 1:**
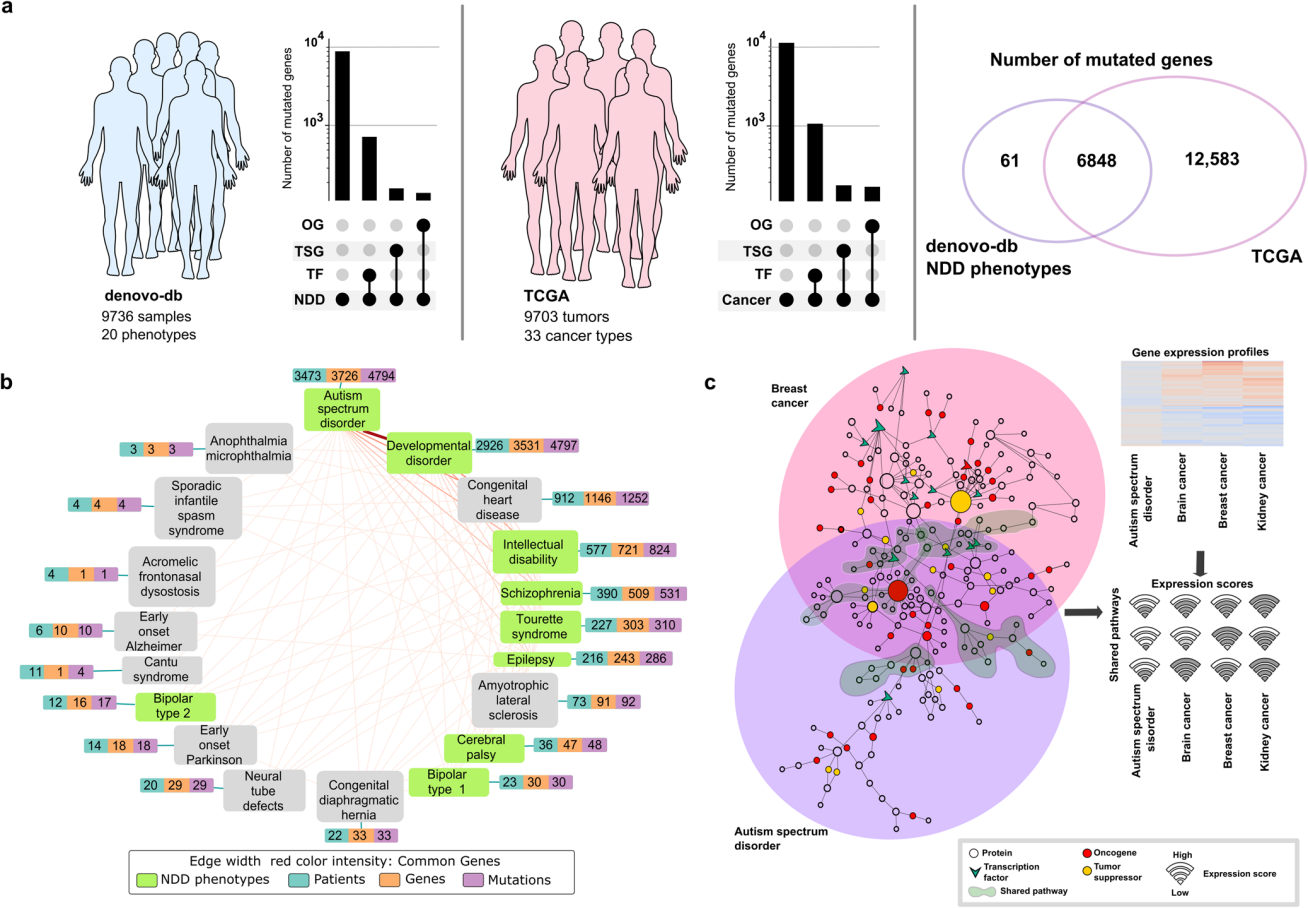
Overview of the data and workflow. **a** Statistics from NDDs and cancer datasets. denovo-db deposits mutation profiles of 9736 samples across 20 phenotypes including eight NDDs (*left panel*). TCGA provides mutation profiles of 9703 tumors across 33 cancer types (*middle panel*). The length of each bar (*y*-axis in a logarithmic scale) in the upset plots shows the number of all mutated genes and the number of TFs, TSGs, OGs among the mutated genes for NDDs (*left panel*) and cancer samples (*middle panel*). There are 712 TFs, 162 TSGs, and 147 OGs out of 7907 mutated genes among denovo-db samples. Similarly, there are 1579 TFs, 259 TSGs, and 249 OGs out of 19,438 mutated genes among the cancer samples. The Venn diagram (*right panel*) shows that there are 6848 common mutated genes between NDDs and cancer; the number of NDD- and cancer-specific mutated genes are 61 and 12,583, respectively. TSG tumor suppressor gene, OG oncogene. **b** Network of phenotypes in denovo-db. Each node represents one phenotype in the network, and each edge represents the connection between two phenotypes if they share at least one commonly mutated gene. NDD phenotypes are shown in green color. Each phenotype is represented with a vector of three numbers; the total number of patients having the phenotype (cyan), total number of genes carrying at least one mutation (orange), and total number of mutations associated with the phenotype (purple). The ticker edges represent the more commonly mutated genes. The most tightly connected pair among the phenotype pairs is autism and developmental disorder. **c** A conceptual representation of network comparison analysis between NDDs and cancer. Two distinct networks (*left panel*) reconstructed for breast cancer (large pink circle) and ASD (large purple circle). These two networks have both shared (shaded green) and separated regions. These networks contain oncogenes (red circle), tumor suppressors (yellow circle), and TFs (green V-shapes). The transcriptome analysis (*upper-right panel*) associates the expression levels of the nodes with the pathway activity. Each enriched pathway in the network can be quantified with the average expression level of its nodes, which is called “pathway scoring.” The score of each shared pathway (1, 2, .., *n*) for each disease (ASD, purple; cancer, red) is calculated (shown as a wifi icon where the higher score is the stronger signal).

To disentangle genetic similarities and differences between NDDs and cancer, firstly we utilized publicly available mutation datasets. Public databases provide somatic mutation profiles of thousands of NDDs and tumor samples, including denovo-db and TCGA, respectively. denovo-db includes de novo mutation profiles for 20 different phenotypes including NDDs and other diseases for 9736 samples^31^; TCGA covers 9703 samples with point mutations across 33 tissues (Fig. 1a). Not all genes and their protein product variants affect the phenotypic output in the same way. Oncogenes, tumor suppressors, TFs, and chromatin remodelers are well-known examples of specific genes whose defects can cause observable alterations in phenotypic outcomes. We compared mutations and mutated proteins between de novo mutations in NDD data deposited in the denovo-db and TCGA, focusing on point mutations that affect only one residue in a protein. We identified 6909 genes in NDDs and 19,431 genes in TCGA with point mutations, among which 6848 genes are common. There are 138 oncogenes, 146 tumor suppressor genes, and 620 TFs in the NDD data, while 248 oncogenes, 259 tumor suppressor genes, and 1579 TFs are in TCGA. ~40% of the mutated genes in TCGA also have mutations in NDD samples.

The network of phenotypes in the denovo-db database covers eight NDD phenotypes including autism spectrum disorder, developmental disorder, intellectual disability, schizophrenia, bipolar disorder, Tourette syndrome, cerebral palsy, and epilepsy and 12 related phenotypes with a varying number of patients, mutated genes, and mutations (Fig. 1b). Only two of these phenotypes—autism and developmental disorders—have more than 1000 samples. In autism, there are 3473 patients, 3726 mutated genes, and 4794 mutations; in the 2926 samples of developmental disorders, there are 3531 mutated genes with 4797 mutations. In the network, the width of edges between the phenotypes is commensurate with the number of commonly mutated genes; autism and developmental disorders share the most. Congenital heart disease and intellectual disability have less than 1000 samples, 912 and 577, respectively. The remaining 15 phenotypes, including schizophrenia, epilepsy, and cerebral palsy, have less than 500 samples.

### Construction and comparison of the networks, expression profiles, and mutation frequencies in NDDs and cancer

Figure 1c outlines our study as follows: First, we reconstructed the PPI networks using mutated genes in breast cancer and ASD as seeds. The networks that we obtained include disease-specific regions as well as shared subnetworks for ASD and breast cancer. Then, we compared the expression scores of the pathways in the shared subnetwork by using gene expression profiles.

Our premise is that NDD mutations offer modest but prolonged signaling, whereas cancer mutations are associated with higher signaling levels^4,45^. Proliferation is a hallmark of cancer. It requires cell growth and division. Proliferation has been associated with multiple dysregulated cellular processes controlling cell life^46^. Key among them are inhibition of cell death, and dysregulation of survival pathways, as in the case of the major MAPK pathway in proliferation, PI3K, Hippo, and Wnt. Differentiation is a hallmark of NDDs. Differentiation is connected to cell lineage. All relate to the cell cycle. Cell cycle dysregulation is a hallmark of tumor cells; cell cycle state regulates differentiation^47^. Signaling that promotes cell proliferation is expected to be stronger than that connected to differentiation.

**Fig. 2:**
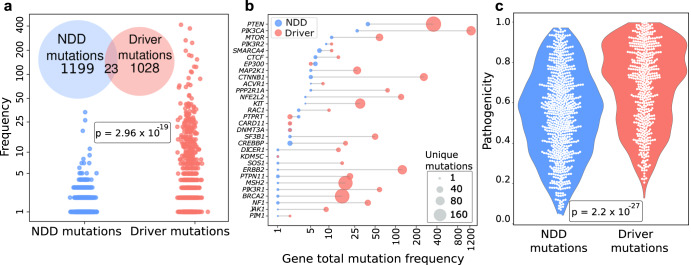
Comparison of mutations between NDDs and cancer. **a** Frequency-based analysis of mutations for NDDs and cancer. The cancer driver mutations in TCGA in comparison to the frequency of NDD mutations. The cancer driver mutations were selected amongst tumor samples only. Among the cancer mutations in TCGA, 23 mutations are shared between NDD and known cancer driver mutations, while 1199 are NDD-specific and 1028 are cancer-specific mutations (inset Venn diagram). Comparison of the frequency of these mutations in the TCGA cohort (*y*-axis in a logarithmic scale, where *frequency* *=* *log10(N* + *1)* and *N* is the number of patients). The difference between mutation frequency distribution in TCGA with *t*-test shows that the mutations present in both NDDs and TCGA are significantly rare in the TCGA cohort when compared to driver mutations (*p* < 0.001). **b** Frequency of mutations on common genes in NDDs and known cancer drivers datasets. The dumbbell plot shows the mutation frequencies of common genes–the genes harboring at least one point mutation among NDDs and cancer samples-in cancer (TCGA) and NDDs (denovo-db) simultaneously. Cancer driver mutations (red) are more frequent than or equal to NDD mutations (blue) except *EP300* and *PTPRT*. The size of the circles represents the number of unique mutations each gene carries. The *x*-axis in a logarithmic scale represents the number of patients having at least one mutation in the corresponding gene in TCGA or NDD sets. **c** MutPred2 pathogenicity scores of NDDs and cancer driver mutations. Violin plots show the distribution of NDD and driver mutation pathogenicity scores. A comparison of the pathogenicity scores using a *t*-test shows that the pathogenicity of driver mutations is significantly higher (*p* < 0.001). Pathogenicity scores are between 0 and 1, where 1 is the most pathogenic.

Driver mutations are frequent, which is why they are often identified as drivers unless there is experimental data for potent rare mutations^45,48^. Weaker or moderate mutations occur less frequently; otherwise, they are drivers. Similarly, the difference between passenger and driver mutations is also based on statistics; their counts are low. As one indicator of mutation strength, we compared the frequency of the cancer driver mutations in TCGA and NDD mutations amongst TCGA samples. For cancer driver mutations, we used the Catalog of Validated Oncogenic Mutations from the Cancer Genome Interpreter (CGI)^49^. Only missense or nonsense mutations were included in the analyses, which comprised 3688 driver mutations in 237 genes. Among 11,576 unique NDD mutations, 1222 are in TCGA (Fig. 2a). On the other hand, TCGA harbors 1060 unique driver mutations. Interestingly, only 23 mutations are shared across known cancer driver mutations and NDDs (see the inset Venn diagram of Fig. 2a). This finding suggests that although there are shared mutations between the two pathologies, these mutations tend to be on the weaker side in terms of a driver effect. In addition, compared to driver mutations, the mutations present in both NDDs and TCGA are notably rare in the TCGA cohort, as demonstrated by the difference in the mutation frequency distribution in TCGA with a *t*-test (*p* = 0.001). Therefore, when we limit the mutations to those present in TCGA, only ~1% of NDD mutations are cancer drivers, and they have very low frequencies among TCGA samples. Figure 2b depicts the number of mutated samples in commonly mutated genes among NDDs and cancer. Most commonly mutated genes have more mutation hits at different positions among all cancer samples. Our observations point to only relatively few common NDDs and cancer driver mutations, making it crucial—even if difficult-to understand the mechanisms through which these common mutations impact gene function and disease phenotypes. We used pathogenicity scores from MutPred2^50^, which probabilistically predict the impact of variants on protein structure and function. We anticipate that variants may have an impact on protein structure, which can either stabilize or destabilize the conformation of the protein depending on protein function and disease phenotypes. The more harmful a mutation is, the closer its pathogenicity score is to one. A comparison of the distribution of the pathogenicity scores of the NDDs and driver mutations calculated using MutPred2 demonstrates that driver mutations have higher pathogenicity than NDD mutations (*t*-test, *p* < 5 × 10^−27^) (Fig. 2c). We observe that most driver mutations accumulate in regions where the pathogenicity scores are larger than 0.8 on the *y*-axis. NDDs harbor mutations in key cancer genes such as *PTEN*, *PIK3CA*, *MTOR*, *KIT*, etc. These mutations have lower frequencies among tumor samples from TCGA, which is an indicator of the lower potency of these mutations. The number of residues hit by mutations among NDD samples is usually lower.

### Distribution of the locations of NDD and cancer mutations and modes of action

**Fig. 3:**
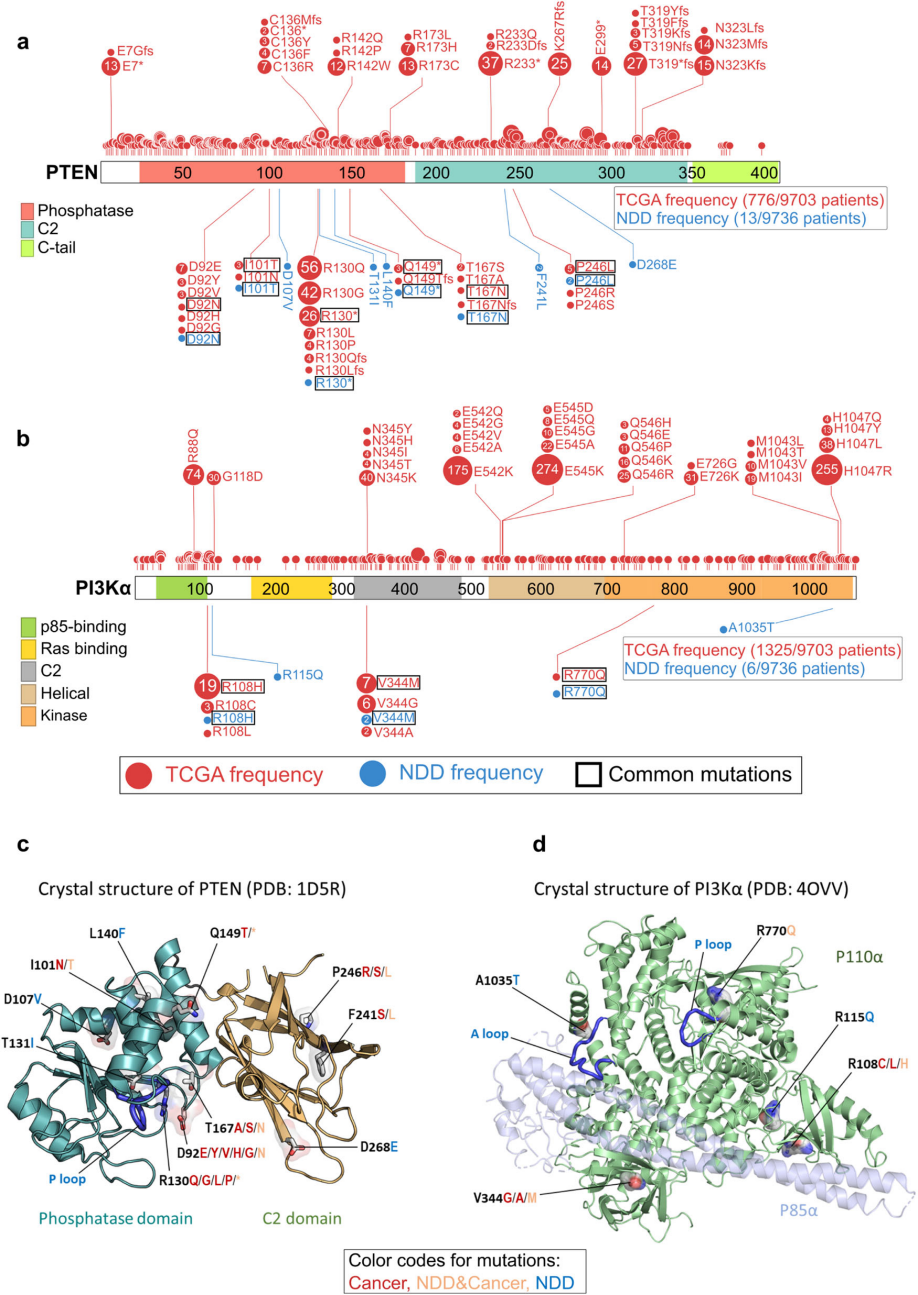
Profiles of TCGA and NDD mutations for PTEN and PI3Kα at the residue level on the sequence and structure. **a** Mutations of PTEN are shown as circles, where the phosphatase domain (red), C2 domain (dark green), and C-tail (light green) are represented as colored boxes along the sequence. The number and size of the circle represent the frequency of each mutation in the NDD (blue) or TCGA (red) datasets. Mutations shared by both datasets are highlighted with rectangular borders for emphasis. Total mutation frequencies and the total number of patients in each dataset are shown in the bottom right box. Nonsense mutations are abbreviated with star (*) sign. 6 of 11 PTEN mutations in the NDD set are present in TCGA. Only R130* has a high frequency relative to other shared mutations, yet it is much less frequent when compared to two other TCGA mutations on the same position, R130Q and R130G. **b** Mutations of PI3Kα (*PIK3CA*) are shown as circles where ABD (green), RBD (yellow), C2 domain (gray), helical domain (light orange), and kinase domain (orange) are represented as colored boxes along the sequence. The number and size of the circle represent the frequency of each mutation in the NDD (blue) or TCGA (red) datasets. Mutations shared by both datasets are highlighted with rectangular borders for emphasis. Total mutation frequencies and the total number of patients in each dataset are shown in the bottom right box. Three out of five PI3Kα mutations in the NDD set are present in TCGA. None of these TCGA mutations are on the most frequently mutated residues or among the most frequent mutations. ABD adapter-binding domain, RBD Ras-binding domain. The 3D structures of **c** PTEN (PDB: 1D5R) and **d** PI3Kα (PDB: 4OVV) with selected NDD and TCGA mutations. For each residue, mutated amino acids are colored in red, blue, or orange if they are present only among cancers, NDDs or both phenotypes, respectively. In PTEN, these mutations are known to affect the functions of protein including loss of phosphatase activity, reduced protein stability at the membrane, and failing to suppress AKT phosphorylation. In PI3Kα, these mutations may interrupt protein activation and reduce protein stability at the membrane.

Phosphatase and tensin homolog (PTEN) and PI3Kα lipid kinase are respectively negative and positive regulators in the PI3Kα/AKT/mTOR pathway. Since the PI3Kα/AKT/mTOR pathway is one of the primary regulators of cell proliferation and differentiation, the mechanistic hallmarks of the mutations are vital to understand. Analysis of mutations in PTEN (Fig. 3a) and PI3Kα (Fig. 3b) sequences reveals that NDD mutations on these proteins usually occur at less frequently mutated sites among tumors (see “Methods”). R130* mutation in NDDs on PTEN is an exception, yet it is less frequent compared to the R130Q and R130G mutations at the same position in cancer.

While several residues of PTEN were mutated in both NDDs and cancer, some mutations—such as T131I, L140F, and D268E—are NDD-specific (Fig. 3a). As to the domain distribution, among the NDD samples, mutated residues D92, I101, R130, T131, L140, Q149, and T167 are on the phosphatase domain, and F241, P246, and D268 are on the C2 domain (Fig. 3c). PTEN’s catalytic activity occurs in the phosphatase domain that contains the P loop (residues 123–130) with the catalytic signature motif, _123_HCxxGxxR_130_ (where x is any amino acid). PTEN mutations in the P loop, or nearby, such as at the residues R130 and T131, can directly constrain the P loop, leading to silencing PTEN catalytic activity. The mutation at residue D92 in the WPD loop (residues 88–98) can disrupt the closed WPD loop conformation that can bring D92 to the active site. D92 is involved in the catalytic activity during the process of hydrolysis to release the phosphate group from Cys124 after transferring it from PIP_3_. Other PTEN mutations, which are distant from the active site, can allosterically bias the P loop dynamics, reducing protein stability and its catalytic activity. A similar pattern is observed in PI3Kα; the rare mutations R108H, V344M, and R770Q are harbored in both NDDs and cancer, while R115Q and A1035T are specific to NDD samples (Fig. 3b). V344 is on the C2 domain; R770 and A1035 are on the N- and C-lobes of the kinase domain, respectively (Fig. 3d). R770 is located near the P loop, and R108 is on the interface of the catalytic subunit p110α and the regulatory subunit p85α. The mutations at these positions in PI3Kα may promote protein activation and increase protein stability at the membrane, but their mutational effects appear to be weaker than the driver mutations.

Several studies investigated germline mutations in PTEN and their association with tumor susceptibility or developmental disorders^51–54^. Although available data are limited, PTEN retains its tumor suppressive function in NDDs while becoming fully dysfunctional in cancer samples.

### NDD- and cancer-specific networks regulate common pathways with different signaling outcomes

**Fig. 4:**
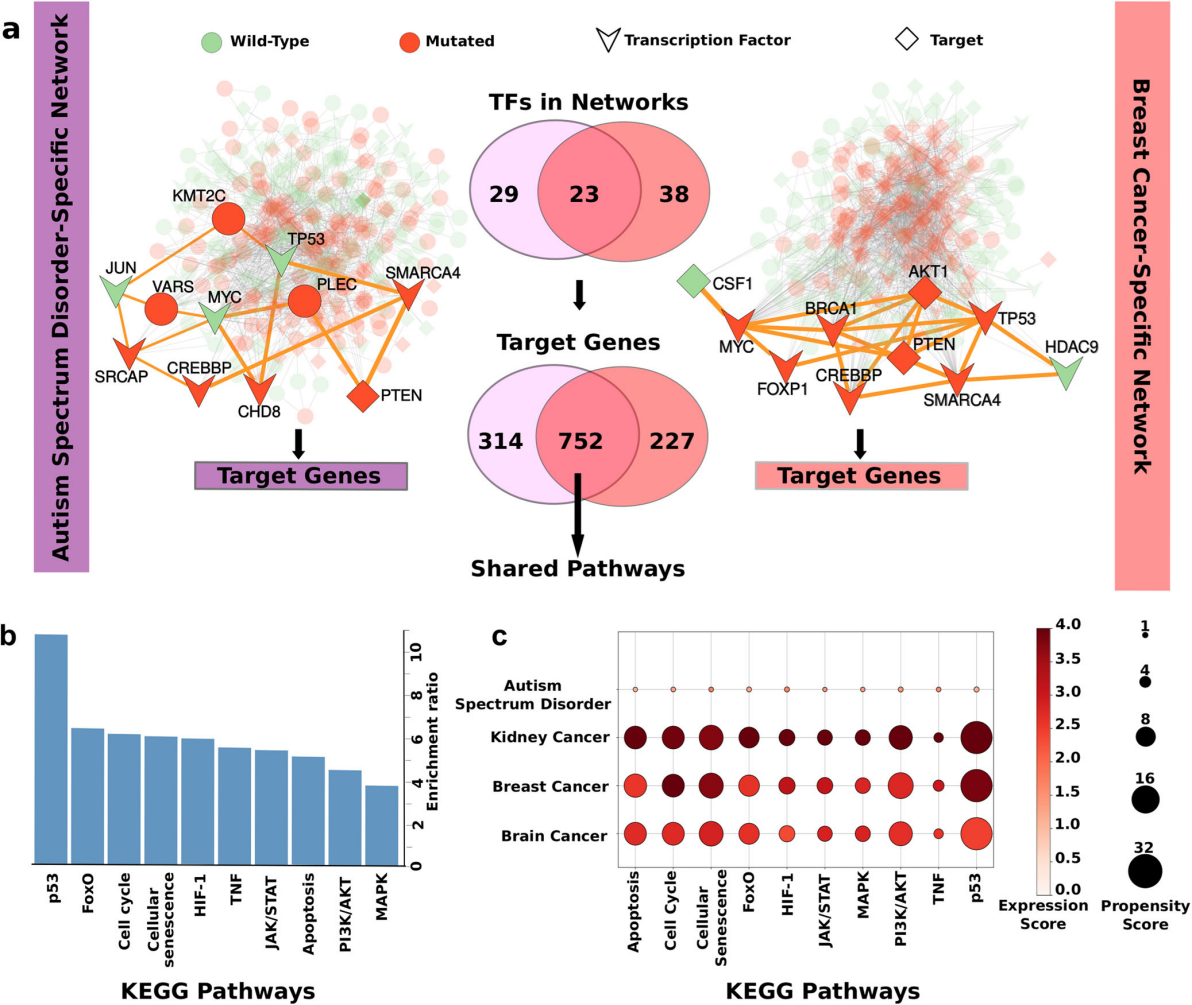
ASD- and breast cancer-specific networks regulating common pathways. **a** Disease-specific network reconstruction for ASD and breast cancer is performed by using pyPARAGON tool, where the frequently mutated genes are used as seeds. The nodes in reconstructed networks involve wild type (green circle), mutated genes (red circle), TFs (chevron), and TF-targets (diamond). The complete ASD-specific network (left side) features the mutated proteins (SRCAP, BRG1, PTEN, etc.) in ASD cases and reveals disease-associated proteins (Jun, p53, and Myc). The breast cancer-specific network (right side) illustrates driver genes, although some driver genes, such as *TP53* and *MYC*, are not frequently mutated in ASD. Both ASD- and breast cancer-specific networks involve 23 common TFs targeting 752 common genes. These common targets are employed to identify shared pathways. BRG1 brahma-related gene 1, a.k.a. SMARCA4, SRCAP SNF2-related CREBBP activator protein, CREBBP cAMP response element binding protein, CHD8 chromodomain helicase DNA-binding protein 8, CSF1 macrophage colony-stimulating factor 1, HD9 histone deacetylase 9, FOXP1 forkhead box protein P1. **b** Overrepresentation analysis determines significant shared pathways (FDR ≤ 0.05) related to cell differentiation and proliferation among KEGG pathways. The pathways include MAPK, PI3K/AKT, and JAK/STAT. These shared TF-target genes play a significant role in cell fate by altering the signal strength and flow, as well as cell cycle and cellular senescence. HIF-1 hypoxia-inducible factor 1, TNF tumor necrosis factor. **c** Signal changes in shared pathways are illustrated with the expression scores of pathways, the mean of the absolute *z*-scores of proteins in a given pathway. We define expression scores as a mean of the absolute *z*-scores of proteins in a given pathway to indicate the magnitude of the deviation from the average expression values of the normal samples, regardless of the direction of the change. The vulnerability of common pathways to mutation is measured with a propensity score, the average unique mutation in the pathway. The darker red represents a higher change in expression scores of genes in the pathway, and the larger circle shows a higher mutation propensity for the corresponding pathway. ASD has the most minor signal differences and mutation propensities compared to all cancer types in shared pathways, where kidney cancer has the highest signal difference. However, there is an insignificant difference in mutation propensities amongst cancer types. The higher expression scores in cancer types point to stronger signal changes in pathways critical for cell fate, such as proliferation and differentiation. The higher propensity scores in cancer reveal that cancer mutations tend to group in shared pathways. Thus, shared pathways are more vulnerable to cancer than ones in ASD. However, mutation loads and signal deviations on the shared pathways might make ASD patients more fragile to cancer onset.

Although alterations in the shared pathways and proteins contribute to the emergence of NDDs and cancer with different weights, the timing of the mutations, the number of activated molecules, the expression level of the mutated protein, and the proteins in the corresponding pathway have a major impact on the phenotypic outcome^4,21^. To understand the divergence between these two pathologies, we analyzed NDD- and cancer-specific networks and compared the signaling outcomes of the pathways using gene expression values. We reconstructed ASD- and breast cancer-specific networks with pyPARAGON^55^ based on frequent mutations, comprising 168 driver genes in breast cancer, and 190 mutated genes that are present in at least three ASD patients. We extracted the graphlet motifs, small significant subnetworks, from the reference interactome HIPPIE through mutations with an unsupervised learning approach^56,57^. To select the most relevant interactions in a disease from the graphlet motifs with the PageRank-Flux algorithm, we constructed a ASD-specific network with 350 proteins and 1291 interactions, and a breast cancer-specific network with 284 proteins and 1878 interactions (Supplementary Data) (Fig. 4a)^55,58^. As can be expected based on our relatively weak mutation outcome premise of NDDs, some critical TFs such as Myc, p53, and Jun with cancer driver mutations are not frequently mutated in ASD. However, mutated genes can indirectly regulate these TFs in the ASD-specific network due to the rewiring of the signaling network. We found 23 common TFs in ASD- and breast cancer-specific networks. TF complexes including Myc/Max or Jun/Fos (AP-1, activator protein 1) regulate the expression of numerous target genes downstream the MAPK phosphorylation cascade in signal transduction^59,60^. Complexes composed of common TFs are primarily involved in cell cycle regulation through their targets, such as E2F mediating cyclin-dependent kinases (CDKs) in cell proliferation^61,62^.

All TFs in ASD- and breast cancer-specific networks regulate 752 commonly targeted genes. The disease models in both networks can use different wiring mechanisms to control shared pathways since different TFs control the transcription of the same genes. Overrepresentation analysis of these common targets demonstrated that shared pathways, including p53, FOXO (forkhead box O), PI3K/AKT, MAPK, and JAK/STAT (Janus kinase/signal transducer and activator of transcription) signaling pathways, are regulated by different TFs (Fig. 4b).

### Gene expression and signaling strength point to differentiation in ASD and proliferation in cancer

Following the construction of the networks and identification of the TFs and their targets, we focused on the signal levels in the constructed networks through an analysis based on differential gene expressions from healthy and disease samples (see “Methods”).

It is challenging to determine how this signal alteration affects these common pathways because of multiple molecular functionalities. Thus, we averaged the absolute values of the differential expression of pathway participants and defined them as the expression score of the given pathway to measure the signal strength in these pathways^63,64^. The expression scores of the overrepresented pathways demonstrated that ASD generated significantly lower signal strength than breast, brain, and kidney cancers (Fig. 4c), influencing the cell cycle at the G1 phase. The change in stimulus and feedback loops regulate signaling intensity and duration^65^. Overexpression and multiple mutation combinations on these pathways disrupt cellular processes and can govern disease development.

The expression profiles of ASD in shared pathways emphasize differentiation. Differentiation reduces the proliferative advantage for the cells and increases their resistance to oncogenic mutations^66^. Mutations in ASD are mostly embryonic; they do not accumulate over time as cancer mutations do. The propensity score of pathways, which demonstrates the probabilities of mutations on a gene in a pathway, reveals that mutations in cancer tend to accumulate in these pathways. Shared pathways in ASD do not have high propensity scores. The already existing mutational burden makes ASD patients more susceptible to multifactorial and/or polygenic diseases, like cancer^6,67^. At the same time, their weak/moderate effect can bring about cell cycle arrest and impact the differentiation capabilities of cells.

### TFs regulating common pathways underscore the trends of differentiation in NDDs and proliferation in cancer

**Fig. 5:**
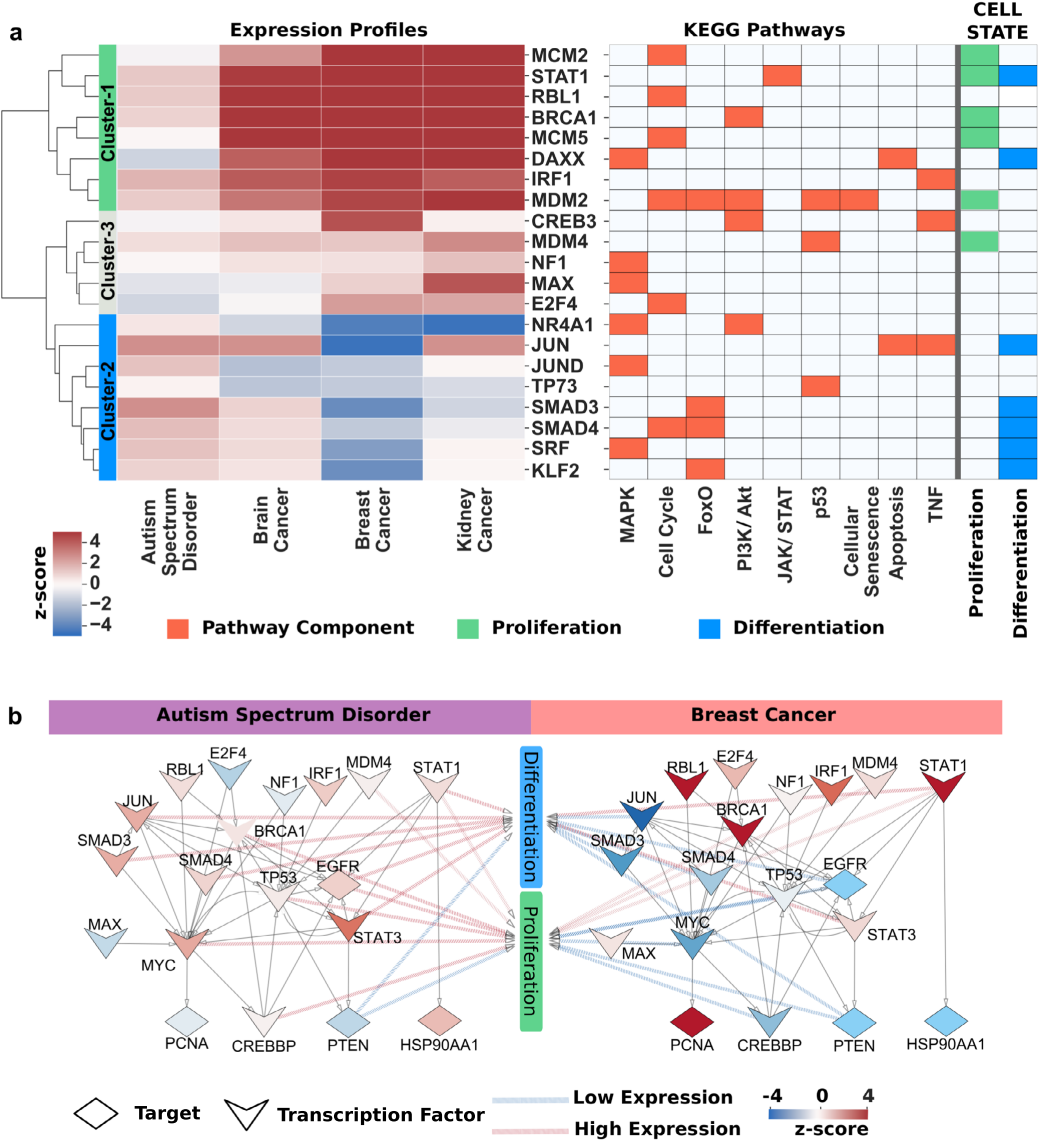
Differential expression profiles in shared pathways. **a** Differential expression profiles of TFs in shared pathways. There were 71 TFs in shared pathways that determine cell fate via changes in signal levels. However, 57 TFs have expression scores in all diseases and 21 TFs were identified to be at least one time differentially expressed more (less) in ASD than in other cancer types. On the left hand, the heatmap of these differentially expressed genes (high in red, low in blue) clustered expression *z*-scores into three groups. On the right hand, the pathways TFs belong to, and related cell states (proliferation, green; differentiation, blue) are demonstrated. *MCM2*, *STAT1*, *BRCA1*, *MCM5*, *DAXX*, *IRF1*, and *MDM2* in cluster-1 are highly expressed in cancers, while *NR4A1*, *JUN*, *JUND*, *TP73*, *SMAD3*, *SMAD4*, *SRF*, and *KLF2* in cluster-2 are highly expressed in Autism. Genes more expressed in cancer types than in ASD mainly belong to the proliferation state, while genes related to differentiation are predominantly more expressed in ASD than in cancer types. **b** Differences between proliferation and differentiation on shared pathways. The signal flows from TFs (chevron) to targets (diamond) in common parts of ASD- and breast cancer-specific networks and in shared pathways were demonstrated with *z*-scores. The low and high expression levels were illustrated with blue to red, respectively. The relationship between cell state and proteins is represented with arrows whose color also demonstrates the level of expressions, low or high. Differentiation-related proteins, such as Jun, SMAD3, and SMAD4, mainly have low expression profiles in breast cancer, while most are highly expressed in ASD. PTEN, EGFR, and STAT1, related to proliferation and differentiation, have similar expression profiles. E2F4 E2F transcription factor 4, RBL1 retinoblastoma-like protein 1, NF1 neurofibromin, IRF1 interferon regulatory factor 1, BRCA1 breast cancer type 1 susceptibility protein, SMAD mothers against decapentaplegic, EGFR epidermal growth factor receptor, PCNA proliferating cell nuclear antigen, CREBBP cAMP response element binding protein, Hsp90α heat shock protein 90α.

For a more in-depth analysis, we compared 71 TFs regulating common pathways through the expression profiles of ASD and breast cancer patients. We observed that 57 TFs have the expression score in ASD, and 21 TFs have distinct expression profiles in ASD and breast cancer that are clustered into three groups (Supplementary Data). Cluster-1 and Cluster-2 demonstrated a distinct separation, while Cluster-3 includes genes that do not show a clear difference in the heatmap of gene expressions (Fig. 5a). The genes in Cluster-1, such as *MCM2*, *STAT1*, *BRCA1*, and *MCM5*, are overexpressed in the cancer samples. These genes mostly play a role in cell proliferation, and their overexpression in cancer promotes cell division and growth^68–71^. On the contrary, ASD samples have relatively lower expression levels for TFs that control cell proliferation. *STAT1* has dual roles in both differentiation and proliferation; it also acts as a tumor suppressor and an oncogene in cancer. The genes in Cluster-2, such as *JUN*, *SMAD3*, *SMAD4*, and *KLF2*, play a role in cell differentiation^72–75^. Their moderate expression levels in ASD suggest that they can maintain the cell differentiation state. To reveal the signal flow starting from these TFs, we defined the regulatory interaction in common pathways by identifying target genes of these TFs. Since one TF can also target other TFs in the same pathway, we extended the regulatory interactions with targeted TFs and their targeted genes (Fig. 5b). Expression profiles of differentiation and proliferation appear moderate in ASD, which suggests weak signal activation in cell proliferation^23^. However, the suppression of differentiation and the overexpression of proliferation indicate strong activation of the proliferation state in cancer.

## Discussion

Here, we comprehensively analyzed mutations, transcriptomic data, and PPI networks of NDDs and cancer patients to comprehend why some mutations can promote cancer while others abet NDDs, and why the same mutations can support both phenotypes. We surmised that cancer mutations are connected to elevated signaling levels, measured by high expression in the shared pathways, while NDD mutations encode sustained but low levels. We further surmised that signaling levels are largely determined by the total number of molecules that the mutations activate, either alone or in combination, along with the cell-type-specific expression levels of the mutant protein and other proteins in the relevant pathways, the timing of the emergence of the mutation (inherited or during embryonic development, or sporadic), as well as additional factors^4^. Ample data indicate that even high expression levels of an unmutated protein can already provoke cancer.

Cancer involves uncontrolled cell proliferation, whereas NDDs are connected to anomalies in the development of the nervous system. Proliferation and differentiation take place in both cancer and NDDs. Since NDDs are mostly related to dysregulated differentiation, mutations in genes regulating chromatin organization rank high. Risk genes for NDDs include more than a third of the cancer driver genes, and NDDs and cancer share hallmarks of cell division and growth^76,77^, thus proliferation and differentiation^7^. In brain cells, embryonic mutations in both pathways give rise to NDDs^78^. Hundreds of genes are implicated in NDDs; however, they are involved in few conserved pathways regulating transcription, including chromatin accessibility, and synaptic signaling^6,79^. PI3K/mTOR and Ras/MAPK are frequently linked with synaptic dysregulation^79,80^. Proteins in the Wnt, BMP/TGF-β (bone morphogenetic protein/transforming growth factor-β), SHH (sonic hedgehog), FGF (fibroblast growth factor), and RA (retinoic acid) pathways, are also involved in autistic brain development^81^. Gene expression profiles of 22 cancer types and frontal cortical tissues from ASD patients identified similarities in genes and pathways^82^.

The tumor suppressor phosphatase and tensin homolog (PTEN), which carries germline and de novo mutations in NDD patients, is related to cancer and several NDDs, collectively named PTEN hamartoma tumor syndrome (PHTS). The NDDs include phenotypes observed in disorders such as Cowden syndrome (CS), Bannayan-Riley-Ruvalcaba syndrome (BRRS), Proteus syndrome (PS), Proteus-like syndrome (PSL), and ASD. NDDs often overlap mutation-wise and genome-wise^83–85^. Among these, deletions, and duplications of the 16p11.2 region are common. About 48% of deletion carriers and 63% of duplication carriers have at least one psychiatric diagnosis^86,87^. RASopathies, which include Noonan syndrome (NS), cardiofaciocutaneous (CFC) syndrome, neurofibromatosis type 1 (NF1), and Legius syndrome (LS), are NDDs that result from overactivation of the MAPK pathway due to germline mutations and/or overexpression in embryogenesis^88,89^. Their phenotypic overlaps may emerge due to shared proteins/pathways as in the case of *PIK3CA*-related overgrowth spectrum (PROS), PS, and CS which share phenotypic characteristics with RASopathies^90^. The commonality of cancer and RASopathies prompted MEK (MAPK kinase) inhibitors and Ras-targeted therapies for some RASopathies like selumetinib for NF1 patients^89,91–93^.

Although there is a strong association between PTEN germline mutations and cancer–PHTS–they have also been described in patients with ASDs^85,94^. PTEN mutations linked to ASD can lead to an unstable but still catalytically active gene product^95^. C124S, G129R, H118P, H123Q, E157G, F241S, D252G, N276S, and D326N are autism-related; A39P, N48K, L108P, L112P, and R130L are PHTS-related mutations^51^. AKT, downstream of PTEN, signaling was suppressed in all seven ASD-related PTEN mutations where PTEN was affected but functional. On the other hand, AKT phosphorylation was promoted by all five PTEN mutations in severe PHTS cases, suggesting that variants with partial loss of PTEN function are predominant in ASD patients^51^. Thus, catalytically inactive PTEN mutant is connected to tumor phenotypes, partially active PTEN to ASD^96,97^.

Dysregulation of the PI3K/AKT/mTOR pathway is a primary factor in NDDs, including megalencephaly (also known as “large brain”), microcephaly (sometimes known as “small brain”), ASD, intellectual disability, schizophrenia, and epilepsy^98^. Mosaic gain-of-function mutations in the *PIK3CA* gene lead to PROS, with clinical outcomes such as excessive tissue growth, blood vessel abnormalities, and scoliosis^99,100^. Among ~200 individuals with *PIK3CA* mosaic mutations, highly activating hotspot mutations were associated with severe brain and/or body overgrowth, whilst fewer activating mutations were linked to more mild somatic overgrowth and mostly brain overgrowth^101,102^. R88Q, V344M, and G914R mutations were identified in PI3Kα patients with macrocephaly and developmental delay or ASD^103^.

We further pursued the complex relationship between genotype and phenotype by constructing disease-specific networks for ASD and breast cancer. We observed distinct PPIs in shared pathways controlling the cell cycle. These rewired interactions could be a reason why shared pathways have different signal strengths in ASD and breast cancer. Under physiological conditions, MAPK and PI3K/AKT/mTOR pathways coregulate the cell cycle through feedback loops to control cell functions, including growth, division, differentiation, and apoptosis. In cancers, they are frequently hyperactivated^104–106^. The PI3K/AKT pathway is also critical in early embryonic development and maintenance of stem cell pluripotency through inhibition of the MAPK proliferation pathway^107–110^. The strength of the signaling perturbations induced by the mutations is manifested in weak/moderate and strong signaling changes, epitomized by ASD and breast cancer, respectively. Strong signals enhance proliferation, and weak/moderate signals may drive cell cycle exit in differentiation^111^.

The expression scores of TFs were grouped based on proliferation and differentiation. TFs enhancing proliferation were mainly overexpressed in cancers while relatively low-expressed in ASD. Proliferating cells can be more vulnerable to mutations than those differentiating, both since dividing cells have less time to repair DNA damage than quiescent cells, and with more replication cycles, there is a higher chance for mutations^66,112^. As to TFs in the differentiation state, ASD has relatively higher expression profiles, while there are significantly low expression profiles in cancers. In cancers, high expression couples with the accumulation of mutations, cell growth, and metastasis^66^.

TF complexes are primarily involved in cell cycle regulation through their targets, such as E2F mediating CDK that accelerates proliferation^61,62^. In the breast cancer-specific network, CDK4 interacts with MAPK1, JAK3, and p53, promoting proliferation^113^. In the ASD-specific network, TF complexes such as forkhead box protein G1 (FOXG1) and sex determining region Y-box 2 (SOX2), also implicated in microcephaly, play critical roles in lineage determination, neural stem/progenitor cell proliferation, and maintenance of pluripotency^114,115^.

Finally, immunity could be viewed as a common factor in NDDs and cancer^4,23^. Multiple pathways related to immunity can be dysregulated in NDDs due to the coevolution of the immune and nervous systems^116^. Signaling pathways related to immunity, such as Wnt, Notch, JAK/STAT, and Hippo, also play roles in cancer metastasis and drug resistance^117,118^.

It is difficult to find ground truth datasets for precise negative/positive controls in complex diseases such as cancer, neurodegenerative disorders (NDGDs), and neurodevelopmental disorders. Apoptosis^119^, differentiation^81^, and proliferation^113^, respectively, may cause NDGDs, NDDs, and cancer due to the altered signal level in cell cycle check point mechanisms^120^. Epidemiologically, cancer is inversely correlated with NDGDs. As in NDDs, the signaling levels of NDGDs can be low, or moderate^121^. We expect that this could be a reason for the inverse relationship with cancer. The low signaling levels in NDGDs can suppress cell proliferation; and conversely, a preexisting cancer with strong proliferative signaling in old age may suppress the emergence of NDGDs. The time windows of the occurrence differ. NDDs’ signaling takes place as the embryo develops. In contrast, NDGDs are much later in life. At the same time, there is a certain overlap in shared pathways and proteins between NDGDs, cancer and NDDs, and all can experience senescence, in aging, in cancer (OIS, oncogene induced senescence)^122^ and in NDDs^121^. We expect that signaling strength, at either extreme, strong, or weak, may abort the cell cycle, leading to premature exit. Comprehensive analysis and comparison of NDGDs with NDDs and cancer may also clarify the impact on cell differentiation^123^.

In a study of the English population, half of the decedents with intellectual disabilities and cancer were at stage IV when diagnosed^124^. Additional statistics reported that the mortality of cancer patients with intellectual disabilities was reported to be approximately 1.5 times higher than the general population^125^. One of the reasons mentioned is that symptoms suggestive of cancer are not always considered due to a bias toward patients. Alternatively, as we discuss below, the preexisting mutational burden may render NDD patients more vulnerable to cancer^6^, with faster cancer progression and higher mortality. As a result of altered signal strength, cancer initiation and progression may differ in individuals with NDD than in the broad apparent NDD-free population, with different outcomes via common pathways.

Our findings offer a mechanistic interpretation for *PTEN* and *PIK3CA* mutations frequently observed in cancer and NDD samples, which may form the basis for functional and detailed structural analysis, including molecular dynamics simulations^126^. Comparing expression scores of shared pathways by leveraging the transcriptomic profiles of NDDs and cancer samples revealed that NDD samples have higher expression scores for genes functioning in differentiation than proliferation. These findings provide an essential step toward understanding the etiology of the two different pathologies, NDDs, and cancer. Despite having common signaling pathways, their regulation and differences in signal levels enhance different cell states: proliferation for cancer and differentiation for NDDs.

Comparisons of the time windows of NDDs and cancer frequently conclude that while cancer is predominantly caused by somatic mutations and alterations in signaling and transcriptional programs, NDDs are primarily linked to germline mutations that express during embryonic development. A recent study has similarly suggested that mutations in cancer susceptibility genes are not necessarily inherited or somatic; they can also arise throughout embryogenesis as a result of errors occurring during cell division^127^. These *mosaic mutations*, occurring in early embryogenesis, were suspected to be associated with some rare cancers. Genetic changes associated with RASopathies are believed to be often sporadic, not inherited. Along these lines, according to the NCI page^128^, this means that typically multiple family members do not share the same NDDs.

Different from this view, here our thesis is that inherited and de novo mutations (missense or truncation) can be major causes of NDDs such as intellectual disability, ASD, epilepsy^129–132^, and cancer. As in cancer, more than one mutation is required for observable symptomatic NDDs. Our premise is that family members can harbor these NDD germline mutations; however, they are not diagnosed as having the disorder. Their offsprings are, however, already susceptible to it. Individuals with NDDs have a higher probability of developing cancer^25,125,133^, likely due to the preexistence of the mutations in the shared proteins, making them more susceptible. Patients with autism have an increased mutation load in genes that drive cancer. We hypothesize that strong driver mutations in cell growth and division pathways are chiefly responsible for uncontrolled cell proliferation in cancer. NDDs’ weak/moderate strength mutations may be a reason why inherited NDDs have not been identified in a parent while predisposing an offspring to it. An additional mutation promotes NDD clinical manifestation. It may be inherited from the other parent or emerge during embryogenesis. It may also promote cancer by providing companion mutations.

Here, we employed de novo mutations in ~8,000 samples with NDDs from denovo-db and somatic mutations in ~10,000 tumor samples from TCGA. We observed that around 40% of the 19,431 mutant genes in TCGA are also altered in NDD samples. 1222 of the 11,576 distinct NDD mutations are present in TCGA. On the other hand, TCGA contains 1051 distinct driver mutations, whereas known cancer driver mutations and NDD only share 23 mutations. This result suggests that common mutations across the two pathologies do exist, although they are typically less potent than cancer drivers. Especially, PTEN and PI3Kα possess a range of mutations scattered through their protein sequences that are either common or disease-specific. This work argues for the examination of such mutations even in undiagnosed family members and their combination in the offspring. It further supports the consideration of cancer pharmacology in NDD patients.

The innovative concept at the basis of this work is that cell proliferation requires a stronger regulatory signal than cell differentiation, and that this difference may explain how the same genes may underpin both cancer (proliferation) and NDDs (differentiation). With our approach, we find that mutations in NDDs tend to have a weaker functional impact and are more likely to influence differentiation compared to those in cancer, which is intriguing and in line with our hypothesis. A major strength of the study is that it provides a broad overview of the mechanistic similarities and differences between the effects of de novo mutations and somatic cancer mutations.

## Methods

### Data collection and processing

NDD mutations were obtained from denovo-db^31^ which holds a collection of human germlines de novo variants of 20 phenotypes. From these 20 phenotypes, we have selected 8 NDDs including ASD, developmental disorder, intellectual disability, schizophrenia, bipolar disorder, Tourette syndrome, cerebral palsy and epilepsy for downstream analysis based on DSM-5 classifications and literature resources^134–137^. Variants from two ASD studies were collected by targeted sequencing of different patients coming from two different studies, while the remaining datasets come from either whole exome or whole genome studies. The phenotypes, the number of samples, unique mutated genes and unique mutations are given in Fig. 1b. We mapped the genomic coordinates to the proteins to obtain the amino acid changes on the protein level using VarMap^138^. We only kept the point mutations that map to the canonical protein sequences. After these filtering steps, we obtained a total of 11,576 unique mutations on 6909 genes from 7880 samples.

Somatic missense and nonsense cancer mutations were downloaded from TCGA. There are 9703 tumor samples from 33 different cancer types in the annotation file where corresponding protein changes are also present. In total, we have 1,546,652 unique mutations on 19,431 genes. 6848 of these genes are also mutated in the NDD dataset. 12,583 of them are only mutated in TCGA, while there are only 61 genes that are mutated solely in NDDs. Because these datasets are open source, no ethics committee authorization or participant consent was required for their use in this study.

### Cancer drivers

A list of cancer driver mutations was downloaded from the Cancer Genome Interpreter (CGI)^139^, which is available as the Catalog of Validated Oncogenic Mutations on their website. We only used missense or nonsense mutations, resulting in an analysis of 3688 driver mutations belonging to 237 genes.

### Visualization of mutations in protein sequences and 3D structures

We used the ProteinPaint tool^140^ to show NDD and cancer mutations on PTEN and PI3Kα. To map the mutations to the 3D structures of PTEN (PDB: 1D5R^141^) and PI3K (PDB: 4OVV^142^) we used PyMol.

### Expression datasets

We utilized processed RNA expression data from ASD, breast, kidney, and brain cancer samples, listed in the Supplementary Data. The ASD dataset was an integrated dataset from the frontal cortex samples in three studies and covered 34 ASD samples and 130 controls. We employed integrated datasets for breast, kidney, and brain cancers that are composed of 7, 10, and 8 studies, respectively. Differential gene expression meta-analyses scored 3579 genes in ASD and 11,629 genes in cancer cohorts with *z*-scores.

### Pathway and network analyses

#### Inference of disease-specific networks

ASD and breast cancer-specific networks were reconstructed with frequently mutated genes and known PPIs. In cases of observations seen in at least 3 patients, 190 genes were selected as seed nodes in ASD. 168 genes were retrieved from the Cancer Genome Interpreter (CGI) and recruited as the seed nodes of breast cancer^139^. The reference network, HIPPIE v2.3, comprises 19,437 proteins and 779,301 PPIs^57^. Each interaction in HIPPIE was scored with a confidence score that was computationally optimized and weighted by the amount and quality of the experimental evidence of PPI. The open-source network inference tool, available at https://github.com/metunetlab/pyPARAGON, pyPARAGON (PAgeRAnk-flux on Graphlet-guided-network for multi-Omic data integratioN), is used to infer ASD and breast cancer-specific networks in three steps. Firstly, pyPARAGON identified an associated region of the reference network through motifs that are frequent non-isomorphic graphlets composed of 2-, 3-, and 4-nodes. The union of significant graphlet motifs constructs a graphlet-guided network (GGN)^55^. Then, The PageRank algorithm weighted all nodes in a reference network, starting from seed nodes. We used the flux computation to weight the edges^58^. In the last step, highly scored interactions in GGN were assembled in our disease-specific networks. We used pyPARAGON with the following parameters: α = 0.5, where α is the probability of walking to neighbor nodes, *τ* = 0.8, where τ is a scaling factor to select a set of top-ranked edges from GGN. This algorithm stops adding edges when the number of interactions reaches 2000.

#### Identification of common pathways

To understand the common functions of disease networks, overlapping regions of networks were analyzed through TFs, target genes, and their associated pathways. TFs and their targets, retrieved from TRRUST v2, were parsed in disease-specific networks, and TFs in these networks were called specific transcription factors (STF)^143^. The targets of STF were selected as regulated genes by disease-specific networks. These commonly regulated genes among ASD and breast cancer were utilized for overrepresentation analysis on WebGestalt to uncover the common pathways (*p* < 0.05 and FDR < 0.05) using manually curated open-source pathway databases, KEGG and Reactome^144–146^.

#### Pathway assessment metrics

The signal strength and mutation vulnerability of the common pathways were evaluated. The expression level of each gene contributes to the signal deviation in the respective pathway. To measure the expression score (*ES*) of a given pathway, we calculated the average absolute signal differences of a pathway^63,64,147–150^ by applying the Eq. (1),

1 $${{ES}}_{P}=\frac{{\sum }_{k=1}^{n}{\rm{|}}{e}_{k}{\rm{|}}}{n}$$

where *P* = (*G*, *E*, *U*), a pathway composed of genes/proteins (*g*_*1*_, *g*_*2*_, …, *g*_*n*_, ∋ *G*), expression of genes (|*e*_*1*_ | , |*e*_*2*_ | , …, |*e*_*n*_ | ∋ *E*), and the number of unique mutations belonging to genes (*u*_*1*_, *u*_*2*_, …, *u*_*n*_ ∋ *U*). We assessed the mutation vulnerability of a pathway by calculating the propensity score (*PS*) of a given pathway considering the number of unique mutations by using the Eq. (2),

2 $${{PS}}_{P}=\frac{{\sum }_{k=1}^{n}{u}_{k}}{n}$$

where the total number of individual mutations in the pathway was normalized with the number of gene members in the pathway.

### Reporting summary

Further information on research design is available in the Nature Research Reporting Summary linked to this article.

### Supplementary information

Supplementary Data

Reporting summary

## Data Availability

The results shown here are in whole or part based upon data generated by the TCGA Research Network: https://www.cancer.gov/tcga. The list of cancer driver mutations underlying the results presented in the study is available in the Cancer Genome Interpreter (CGI) as Catalog of Validated Oncogenic Mutations: https://www.cancergenomeinterpreter.org/home. The reference PPI network, HIPPIE v2.3 was downloaded from http://cbdm-01.zdv.uni-mainz.de/~mschaefer/hippie/download.php. 3D protein structures were obtained from Protein Data Bank (PDB): https://www.rcsb.org. Data analyzed in this study are available with the accession codes GSE28475, GSE28521, GSE4290, GSE9385, GSE74195, GSE68848, GSE15824, GSE42568, GSE54002, GSE65216, GSE45827, GSE29431, GSE11151, GSE77199, GSE47032, GSE53757, GSE53000, GSE66272, GSE68417, GSE71963, GSE40435, GSE7635 in Gene Expression Omnibus: https://www.ncbi.nlm.nih.gov/geo/. Transcription factors and their targets were retrieved from TRRUST v2: https://www.grnpedia.org/trrust/. Pathways were obtained from KEGG database: https://www.genome.jp/kegg/pathway.html.

## Code availability

All the code used for data analysis and generation of figures will be available upon request. ASD and breast cancer-specific networks were prepared using the open-source network inference program pyPARAGON (PAgeRAnk-flux on Graphlet-guided-network for multi-Omic data integratioN), which is accessible at https://github.com/metunetlab/pyPARAGON.
